# Physical Activity and Diet in a Global Pandemic: An Investigation of the Impact of COVID-19 on Factors Relevant for Musculoskeletal Health at Two Different Stages of the Lifecourse

**DOI:** 10.3389/fendo.2022.882399

**Published:** 2022-05-03

**Authors:** Gregorio Bevilacqua, Stefania D’Angelo, Cathy Linaker, Alice Paul, Ilse Bloom, Jean Zhang, Faidra Laskou, Cyrus Cooper, Kate A. Ward, Karen Walker-Bone, Elaine M. Dennison

**Affiliations:** ^1^Medical Research Council (MRC) Lifecourse Epidemiology Centre, University of Southampton, Southampton General Hospital, Southampton, United Kingdom; ^2^Medical Research Council (MRC) Versus Arthritis Centre for Musculoskeletal Health and Work, University of Southampton, Southampton, United Kingdom; ^3^National Institute for Health and Care Research (NIHR) Southampton Biomedical Research Centre, University of Southampton and University Hospital Southampton National Health Service (NHS) Foundation Trust, Southampton, United Kingdom; ^4^National Institute for Health and Care Research (NIHR) Oxford Biomedical Research Centre, University of Oxford, Oxford, United Kingdom; ^5^School of Biological Sciences, Victoria University of Wellington, Wellington, New Zealand

**Keywords:** COVID-19, musculoskeletal health, diet, physical activity, older adults

## Abstract

**Background:**

Physical activity, nutrition and other lifestyle factors play important roles in maintaining musculoskeletal health. The coronavirus disease (COVID-19) originated in late 2019, spread globally to be declared a pandemic by the World Health Organisation in March 2020, and led to widespread behaviour change. The aim of this study was to use two existing cohorts, the Hertfordshire Cohort Study (HCS) and Health and Employment After Fifty Study (HEAF), to understand how wave one of the COVID-19 pandemic impacted lifestyle factors associated with musculoskeletal health in the UK.

**Methods:**

125 eligible participants, 65 males and 60 females (drawn from the HCS study, median (IQR) age 84.3 (82.4-86.6) years, all Caucasian, and community dwelling) were contacted by telephone and asked to complete a questionnaire administered by a trained researcher. Data collection occurred over the period July 2020 to February 2021. 2469 participants, 1086 men and 1383 women (drawn from the HEAF study, median age 65.7 (62.0-69.3) years, mostly Caucasian and community dwelling) completed an online questionnaire in March 2021.

**Results:**

In HCS, 47% respondents reported being less physically active than before the pandemic (and only 5% more so), 27% said they consumed less alcohol compared to pre-pandemic times (and only 3% more so), and 18% reported eating less than before, although quality of diet was generally unchanged over this timeframe surveyed. In HEAF, 44% participants said they were less active than before the pandemic, while 17% reported being more active. The majority of participants reported no changes in alcohol consumption and diet; however, 19% said they drank more than before (32% of which was above recommended levels), 16% said their diet was less healthy, and 19% reported eating more than before.

**Conclusion:**

We have reported the experience of the first wave of the COVID-19 pandemic among participants of two Caucasian community dwelling UK cohorts, highlighting the impact of the pandemic on lifestyle factors associated with musculoskeletal health. Changed physical activity levels were reported in a high proportion of respondents in both studies; an investigation of reversibility of these changes is required.

## Introduction

The coronavirus disease (COVID-19), caused by the severe acute respiratory syndrome coronavirus 2 (SARS-CoV-2) was declared a pandemic by the World Health Organisation in March 2020. To date, the rapid spread of this virus has led to more than 360 million infections and caused the death of well over 5 million people globally (1). In the early stages of the pandemic, several countries introduced a number of measures and restrictions aimed at reducing human-to-human transmission of the disease; these included social distancing, requirement to stay at home unless an essential worker, closure of schools, curfews and prohibiting social gatherings (2). On the 23^rd^ of March 2020, the United Kingdom entered its first national lockdown: people were required to work from home if possible and socialising was allowed exclusively among members of the same household (3).

Older adults are particularly vulnerable to the COVID-19 virus, with their associated higher risk of severe disease, morbidity and mortality (4, 5). While the social distancing and self-isolation strategies proved successful in reducing numbers of new infections, hospitalizations, and deaths, they may have had an impact on lifestyle factors such as physical activity and diet (2, 6–12), with potentially severe longer-term consequences for cardiovascular and mental health. While some adults may have taken the opportunity to make positive lifestyle changes, others may have been more adversely affected. We hypothesized that different ages of adults may have been affected differently i.e. adults in midlife may have made positive changes, due to working at home or being furloughed and hence having more time, while since older populations were declared as “vulnerable” they may have felt more constrained and their lifestyle might be negatively impacted due to fear of going outdoors (6, 8, 13–17).

Regular physical activity and adequate nutrition are known to be associated with better musculoskeletal health in later life (18–20), and physical activity is essential for older adults to maintain their independence, physical and mental health, and overall wellbeing (21, 22). Given that older adults are at greater risk of more severe disease, and will be aware of this, we hypothesized that the UK lockdown may have had different impacts on lifestyle factors of older adults of different ages. We hypothesised that older adults might be more reluctant to access their usual suppliers of food and may also have curtailed leisure activities that they participated in before the emergence of COVID-19.

In this study, we used data collected during the first wave of the pandemic from two existing cohorts of UK community-dwelling older adults who were at different stages in the lifecourse: the Health and Employment After Fifty Study (HEAF), median age 65 years, and the Hertfordshire Cohort Study (HCS), median age 84 years. Here we report how the first wave of the COVID-19 pandemic impacted on lifestyle factors that are important for musculoskeletal health, and to consider whether the impact was different in the two populations.

## Materials and Methods

The HCS is a population-based sample of men and women born between 1931–9 in Hertfordshire and originally recruited in order to study the relationship between growth in infancy and the subsequent risk of adult diseases (23, 24). Between 2019 and 2020, 176 participants from the HCS were visited at home by trained fieldworkers who administered a questionnaire collecting lifestyle information such as alcohol consumption (measured as units consumed in a week). The visits also included measurements of height and weight to calculate body mass index (BMI). Between July 2020 and February 2021, 125 of these participants (65 men and 60 women) were contacted by telephone and consented to complete an additional questionnaire administered by a trained researcher.

During the telephone survey, participants were asked to evaluate how aspects of their lifestyle had changed since the official lockdown was announced on 23 March 2020 until July 2020. These changes were assessed asking the following questions: “Compared to before the lockdown began, during the lockdown period: How much alcohol did you drink? How much did you eat? How was your diet? How physically active were you?”. Possible answers were: “Less than usual”, “About the same”, “More than usual”, “I do not drink” (for change in alcohol consumption); “Less than usual”, “About the same”, “More than usual” (for change in food intake and physical activity); “Less healthy than usual”, “about the same healthiness as usual”, “More healthy than usual” (for change in diet quality).

The HEAF study is an observational cohort set up in 2013 with the intent to explore the health benefits and risks of extending working lives and conversely to explore the impact of health on employment outcomes. People were recruited when aged 50-64 years and were followed-up annually after the baseline questionnaire. Participants with an email address were sent an online questionnaire in March 2021, in order to explore changes that the first 5 months of lockdown had brought to their lives. Out of 4665 HEAF participants originally contacted, 2469 participants (1086 men and 1383 women) completed the questionnaire.

Among other questions, participants were asked whether they perceived that their lifestyle had changed since the beginning of the March 2020 lockdown, and whether their alcohol consumption, level of physical activity, healthiness of diet and amount of food intake had changed. Participants were also asked to report their weight (either in kg or stones), while height was self-reported during the first pass of the HEAF study (2013–2014); from these, BMI was derived. In both cohorts, weekly alcohol units were derived by adding up the number of glasses of wine, measures of spirits and double the pints of beer/ciders a person self-reported. According to NHS guidelines, to keep with healthy drinking, men and women should not consume more than 14 units or alcohol per week (25).

### Statistical Analysis

Numbers and percentages were used to describe participants by categories of changes in each lifestyle factor. Analyses were conducted for the overall sample as well as stratified by sex. Pearson’s chi-squared, t-test or Wilcoxon rank-sum test were used to compare characteristics between men and women, depending on the nature of the variable. Analyses were performed with Stata statistical software (v 17.0).

## Results

Responses were received from 125 HCS participants [median (IQR) age: 84.3 (82.4-86.6) years] and 2469 HEAF participants [median (IQR) age: 65.7 (62.0-69.3) years].

**Figure 1:**
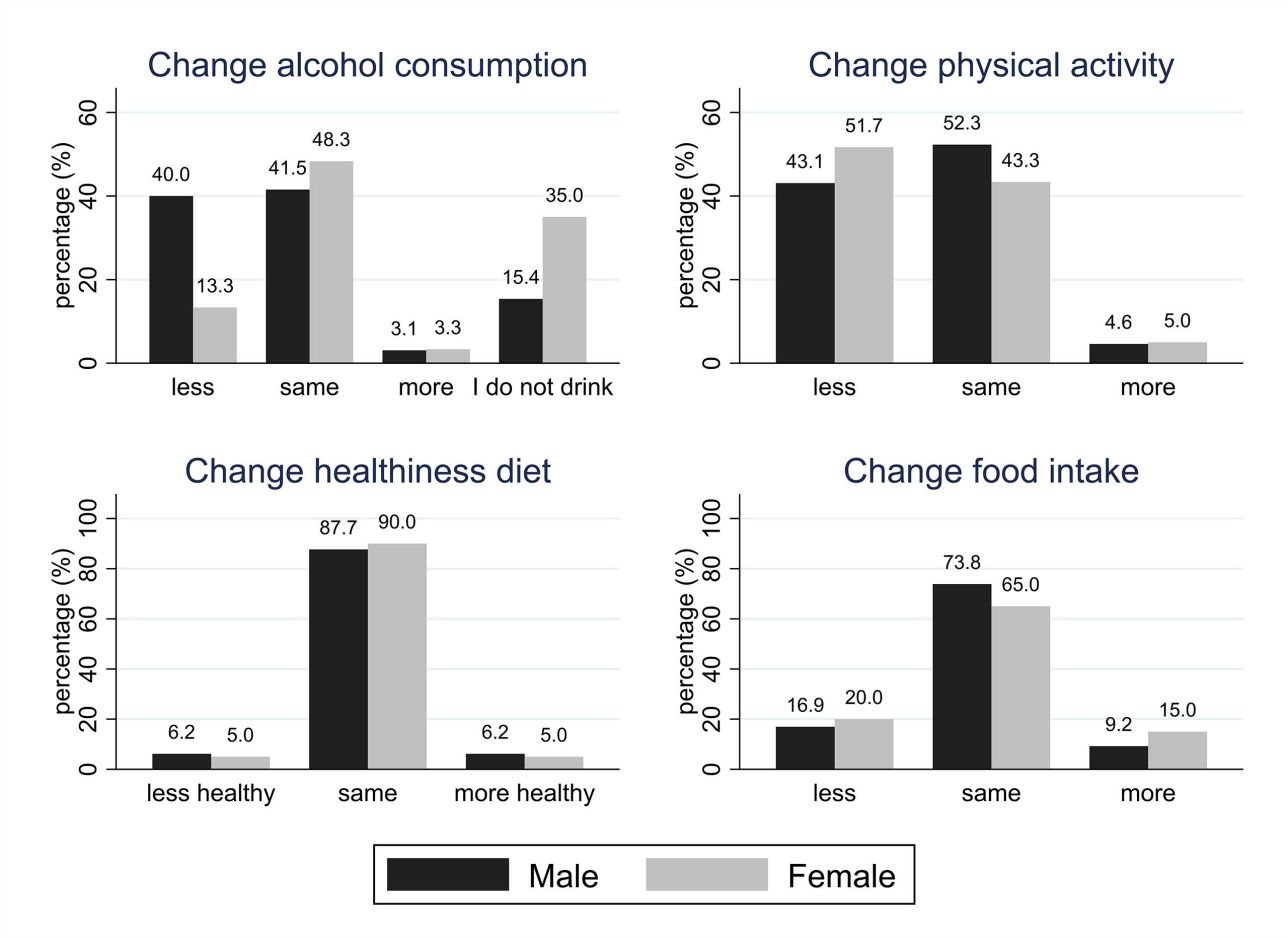
Changes in lifestyle factors in HCS participants.

**Table 1** and **Figure 1** provide the main characteristics of and results for the HCS participants included in this study. In HCS, alcohol consumption (reported during home visits in 2019-2020, shortly before the pandemic) was generally higher among men [median (IQR): 3.0 (0.3-10.9) units/week] than women [median (IQR): 0.8 (0.0-3.8) units/week]. This sex difference was statistically significant. Based on NHS guidelines (25), 18.5% of men and 5% of women reported excessive drinking (>14 units of alcohol per week). More than a quarter of HCS participants (27.2%) reported drinking less alcohol than usual during the pandemic (only 3.2% said that they drank more); however, more men (40%) than women (13.3%) reported that they had reduced their alcohol consumption since the start of lockdown, and this difference was statistically significant (p=0.002). Almost a fifth of HCS participants (18.4%) reported eating less than they did in pre-pandemic times, while 12% said that they were eating more than usual, although the quality of diet remained largely unchanged, with 88.8% of the HCS sample reporting that their diet had been as healthy as usual, and only 7 participants (5.6%) reporting that their diet became less healthy. Less than half of HCS participants said that they were as physically active as usual (48%), while 47.2% reported being less active, and just 4.8% stated that they had become more active than usual. With the exception of changes in alcohol consumption, no statistically significant sex differences emerged. Approximately a third of the overall sample reported a normal BMI (18.5-24.9 km/m2) with no significant difference between men and women.

**Table 1:** HCS participants’ characteristics and self-reported lifestyle changes compared to pre-pandemic times.*

	Overall (n=125)	Men (n=65)	Women (n=60)	p-value
*Age (yrs) (median (IQR))*	84.3 (82.4-86.6)	83.6 (82.3-86.3)	85.1 (82.5-87.7)	0.14
*BMI (kg/m2) (mean(SD))*	27.05 (4.00)	27.17 (3.54)	26.94 (4.47)	0.753
*Normal BMI (18.5-24.9 kg/m2)*	40 (32.8)	18 (28.6)	22 (37.3)	0.51
*Alcohol (unit/week) (median (IQR))*	1.73 (0.1-8.28)	3.0 (0.3-10.9)	0.8 (0.0-3.8)	0.002
*Excessive alcohol (>14 units/week)*	15 (12.0)	12 (18.5)	3 (5.0)	0.02
*Changes to alcohol intake*				0.002
* Less than usual*	34 (27.2%)	26 (40%)	8 (13.3%)	
* About the same*	56 (44.8%)	27 (41.5%)	29 (48.3%)	
* More than usual*	4 (3.2%)	2 (3.1%)	2 (3.3%)	
* I do not drink*	31 (24.8%)	10 (15.4%)	21 (35%)	
*Changes to food intake*				0.502
* Less than usual*	23 (18.4%)	11 (16.9%)	12 (20%)	
* About the same*	87 (69.6%)	48 (73.8%)	39 (65%)	
* More than usual*	15 (12%)	6 (9.2%)	9 (15%)	
*Changes to healthiness of diet*				0.92
* Less healthy than usual*	7 (5.6%)	4 (6.1%)	3 (5%)	
* About the same healthiness*	111 (88.8%)	57 (87.7%)	54 (90%)	
* More healthy than usual*	7 (5.6%)	4 (6.1%)	3 (5.6%)	
*Changes to physical activity*				0.57
* Less than usual*	59 (47.2%)	28 (43.1%)	31 (51.7%)	
* About the same*	60 (48%)	34 (52.3%)	26 (43.3%)	
* More than usual*	6 (4.8%)	3 (4.6%)	3 (5%)	

*All percentages are based on non-missing data for each variable.

**Figure 2:**
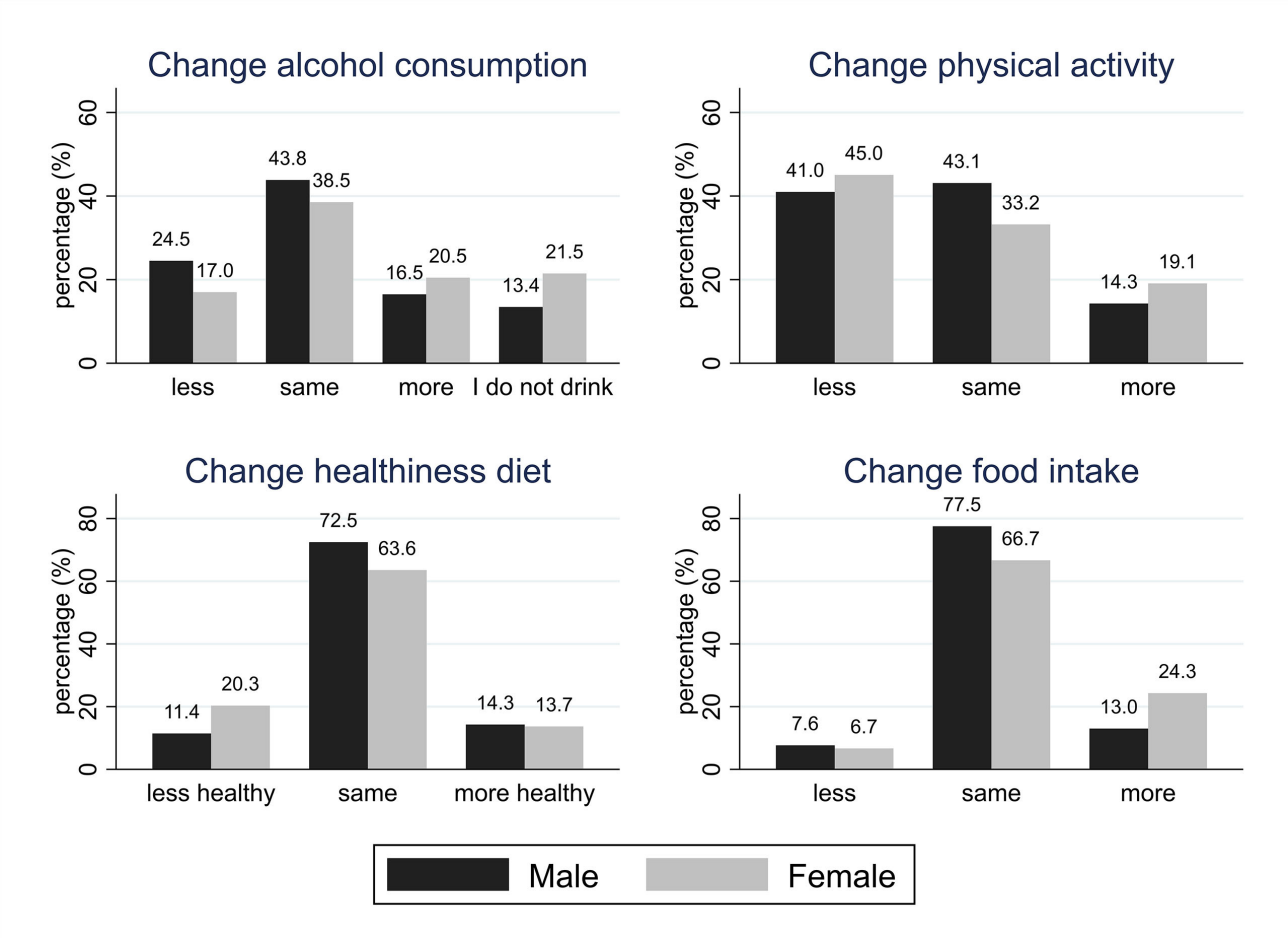
Changes in lifestyle factors in HEAF participants.

Characteristics and results for HEAF participants are reported in **Table 2** and **Figure 2**. Male HEAF participants had a higher BMI [mean (SD): 27.3 (4.9) kg/m^2^] than women [mean (SD): 26.5 (5.6) kg/m^2^] and consumed mo+re alcohol [median (IQR): 8.00 (2.5-14.0) units/week] than female participants [median (IQR): 4.00 (0-8.0) units/week]; both differences were statistically significant. Approximately 23% of men and 7% of women reported excessive drinking. Most participants (41.8%) reported drinking the same amount of alcohol as they used to before the pandemic, although almost a fifth (19.1%) said they were drinking more than usual (32% of whom were now drinking above recommended low-risk levels, defined as 14 units per week (25). More HEAF women (21%) than men (16.8%) said that they had increased their alcohol consumption during the first wave of the pandemic, and the difference was statistically significant (p<0.001). Slightly less than a fifth (19.7%) of HEAF respondents reported that they had been eating more than usual since the first UK-wide lockdown was announced, with a statistically significant difference between women and men (24.9% and 13.2% respectively, p<0.001). Overall, 16.8% reported that their diets were less healthy than pre-pandemic times, and this difference was more commonly reported by women than men (20.8% and 11.6% respectively, p<0.001). In HEAF, 44.2% of respondents said that they were less physically active than before the pandemic, while 17% reported having been more active. More women (46.3%) than men (41.7%) reported being less active, although there were also more women (19.6%) than men (14.5%) saying they became more physically active, with this difference being statistically significant (p<0.001).

**Table 2:** HEAF participants’ characteristics and self-reported lifestyle changes compared to pre-pandemic times.*

	Overall (n=2,469)	Men (n=1,086)	Women (n=1,383)	p-value
*Age (yrs) (median (IQR))*	65.6 (4.3)	65.9 (62.1, 69.5)	65.4 (61.8,69.1)	0.01
*BMI (kg/m2) (mean (SD))*	26.9 (5.3)	27.3 (4.9)	26.5 (5.6)	<0.001
*Normal BMI (18.5-24.9 kg/m2)*	929 (39.3)	340 (32.4)	589 (44.8)	<0.001
*Alcohol (unit/week) (median (IQR))*	5.0 (1.0,11.0)	8.0 (2.5,14.0)	4.0 (0,8.0)	<0.001
*Excessive alcohol (>14 units/week)*	341 (14.4)	246 (23.3)	95 (7.3)	<0.001
*Changes to alcohol consumption*				
* Drunk less*	501 (20.8)	266 (24.9)	235 (17.4)	<0.001
* Drunk about the same*	1009 (41.8)	476 (44.6)	533 (39.5)	
* Drunk more*	462 (19.1)	179 (16.8)	283 (21.0)	
* Do not drink*	443 (18.3)	146 (13.7)	297 (22.0)	
*Changes to food intake*				
* Less than usual*	175 (7.3)	83 (7.8)	92 (6.8)	<0.001
* About the same*	1764 (73.0)	842 (79.0)	922 (68.3)	
* More than usual*	477 (19.7)	141 (13.2)	336 (24.9)	
*Changes to healthiness of diet*				
* Less healthy*	405 (16.8)	124 (11.6)	281 (20.8)	<0.001
* About the same*	1666 (69.0)	787 (73.8)	879 (65.2)	
* More healthy*	344 (14.2)	155 (14.5)	189 (14.0)	
*Changes to physical activity*				
* Less active*	1068 (44.2)	445 (41.7)	623 (46.3)	<0.001
* Equally active*	927 (38.4)	468 (43.8)	459 (34.1)	
* More active*	419 (17.4)	155 (14.5)	264 (19.6)	

*All percentages are based on non-missing data for each variable.

When we compared the self-reported lifestyle changes across the 2 cohorts, we found that they were mostly significantly different between HEAF and HCS.

## Discussion

We have reported how two cohorts of community-dwelling older UK adults (mostly Caucasian) described the effects of the first wave of the COVID-19 pandemic on their lifestyle, focussing on health behaviours relevant to musculoskeletal health. Both cohorts reported important effects of the lockdown on these lifestyle factors, specifically on levels of participation in physical activity. However, along with a number of similarities, we noticed some differences between the younger (HEAF) (median age 65 years) and the older (HCS) (median age 84 years) cohorts, such that the younger adults reported drinking more alcohol, with a significant number reporting drinking in excess of weekly drinking guidelines for the first time; there were also higher proportions of HEAF participants reporting changes in diet quantity than before the pandemic, compared to the older cohort. Within HEAF, women reported a number of changes of concern: higher proportions of women reported consuming more alcohol and food and reducing the healthiness of their diet since the pandemic.

Excess alcohol consumption is detrimental to both muscle and bone health; drinking to excess is associated with lower bone density (26–29), more falls, and poorer muscle quality (26, 30–34). Consequently, excess alcohol consumption is associated with an increased risk of fracture. In both cohorts, there was a significant sex difference in alcohol consumption, with men drinking more alcohol units per week than women. A recent cross-country study by Calvo et al. using data from the English Longitudinal Study of Ageing (ELSA), showed that alcohol consumption remained higher in men than in women older than 65 years, with men having almost twice the number of drinks per day than women (35). It is noteworthy that, in both our cohorts, more men than women reported that, since the pandemic, they consumed less alcohol than they used to. Similar findings were reported in a cohort of adults from the United States of America, mean (SE) age 67.7 (0.2): in this population, fewer women (46.5%) than men reporting reducing their alcohol consumption since the beginning of the pandemic (36). The COVID-19 pandemic has obviously changed the contexts in which both men and women consume alcohol, essentially reducing available drinking locations to a single one: one’s own home. In this respect, a study conducted with US and Canadian adults reported that, although men drank more than women, the difference was more noticeable when alcohol was consumed in social contexts such as at home with visitors and in bars (37). It is possible that, in our cohorts, the lack of a social context in which alcohol could be consumed, due to the pandemic, may have influenced the reduction of alcohol drinking in men. We noticed that almost a fifth (and mainly women) of the younger cohort said that they had started drinking more since the start of the pandemic. Recent studies suggest that female sex could be a predictor for negative psychological impact during the COVID-19 pandemic (38, 39), and therefore women may have been more inclined to resort to alcohol in order to cope with the stressful context of this global pandemic (40).

Nutritional factors are very important for musculoskeletal health; factors associated with better bone health include an adequate intake of calcium, protein and other vitamins and minerals (41–45). Several studies have highlighted the importance of adequate intake of protein for muscle health (46–50). In both HCS and HEAF, the majority of participants said that they did not change the quantity of food during lockdown relative to what they used to eat before the pandemic, although our findings suggested that older adults were more likely to decrease their food intake and younger adults were more likely to increase it, particularly younger women. The majority of participants from both cohorts also reported that the healthiness of their diet remained unchanged, although almost 17% of HEAF participants declared that their diet became less healthy since the start of the pandemic, with significantly more women reporting this change. These preliminary findings warrant greater investigation to understand what changes have occurred, and whether they have been reversed with time, emergence from lockdown and a move to the ‘new normal’. Reduced intake of calcium rich foods, for example, might be particularly detrimental to women who are recently postmenopausal. Our findings are in accord with two recent studies that found that diet quality and food intake were mainly unaffected during the lockdown among older populations (51, 52).

Of some concern, we found that a higher proportion of HCS participants reported that they were eating less than before the pandemic. Being notably older than HEAF participants, HCS respondents may have been experiencing more functional limitations and frailty (52), and therefore might have experienced more difficulties in preparing meals at home and/or procuring themselves certain foods, with some of them probably relying on family and/or friends for grocery shopping and meal preparation. Malnutrition is recognised as a significant issue in many older adults (53–55) and is associated with a number of adverse health outcomes (53, 56). For this reason, these observations are of concern, and warrant follow up. Investigation of factors leading to these changes, and understanding whether and how they are reversible, will be important, especially for future pandemic management.

Lastly, in both cohorts, a greater proportion of participants reported that they were doing less physical activity during the pandemic, although the pattern was more mixed in midlife, where women in particular were more likely to have changed physical activity patterns. This is of great importance as regular weight bearing activity is associated with better bone health (57–59) and resistance exercise is associated with benefits for muscle (60–62). It has been previously reported that being socially isolated is associated with decreased physical activity (63–65). Importantly of course, gyms and leisure centres were closed and group activities such as yoga and walking football became unavailable. Our findings on physical activity are in line with a number of studies conducted worldwide, which reported a general decrease in active time during the pandemic (2, 7, 66–69). The fact that, compared to HEAF, more participants from our older cohort decreased their physical activity is most likely related to pre-existing low levels of physical activity: a study conducted with older adults from New Zealand by Mummery et al. compared physical activity in two age groups (60-64 years old and 80+ years old) and found that physical activity in the older group declined by more than 24% (70). The greater decline in physical activity in HCS can be ascribed to the likelihood that, due to age and perhaps comorbidity, HCS respondents were experiencing more functional limitations and frailty (52), and that the restrictions imposed on them by lockdown measures may have exacerbated such limitations. In addition, group activities previously attended by older adults (e.g., social indoor/outdoor games, social clubs, courses and classes, volunteering) became unavailable due to pandemic-related restrictions. This is also in line with the finding of a study by Cancello et al., conducted among a population sample of Italian adults (20% of which were older than 60 years): this study found that age was one of the main determinants of lifestyle changes during the pandemic (66), although pre-lockdown exercising habit was a fundamental determinant of lifestyle changes during the pandemic (with 68% of participants who were active before lockdown decreasing their exercise, and 27% of participants who were sedentary before the pandemic starting to exercise), while other factors such as BMI and sex were not (66).

Our study has a number of limitations. The responder sample of the HCS may not be representative of the wider UK population aged 84 years, since the sample consists only of 125 individuals and all recruited participants were born in the county of Hertfordshire, were still living in their homes, had survived to age > 80 years and were all Caucasian (23). However, it has been previously demonstrated that the cohort from which the respondents were drawn is representative of the general population with regard to anthropometric body build and lifestyle factors, such as alcohol intake, in line with data found in the European Investigation into Cancer and Nutrition Cohort (EPIC) (71). Likewise, HEAF obtained a 20% response rate at baseline and responders were mostly Caucasian and tended to be somewhat older, more affluent, and more likely female than non-responders (72). However, they were geographically diverse and represent every decile of deprivation. Changes in lifestyle factors were self-reported in both cohorts and therefore recall bias cannot be ruled out. Our measures were fairly blunt, and were collected at slightly different phases of the pandemic, for example single questions around level of physical activity rather than specifics of how activity levels or diet had changed. Lastly, while HEAF included a relatively large sample of participants, the number of HCS participants in this study was much lower, and this may explain why we did not find many statistically significant sex differences in this older cohort. In future, we plan to investigate the reversibility of the lifestyle changes we have observed and the drivers behind them, specifically changes in working patterns in younger adults.

## Conclusions

In this study, we have reported how UK adults in midlife and later life reported changes in lifestyle factors in the first wave of the COVID-19 pandemic. We found some self-reported changes in alcohol intake and eating habits in both groups, with potentially harmful changes taking place more frequently in the younger cohort than in the older, and more notably among women. Physical activity levels decreased for high proportions of respondents in both cohorts. While the aim of this study was mainly descriptive, our findings suggest that age and sex might play important roles in the impact of the COVID-19 pandemic and subsequent lockdown on lifestyle factors.

## Data Availability Statement

The HCS dataset used and/or analysed during the current study are available from the corresponding author on reasonable request. The HEAF dataset used and/or analysed during the current study are available on reasonable request from the “MRC Versus Arthritis Centre for Musculoskeletal Health and Work” by contacting Karen Walker-Bone (kwb@mrc.soton.ac.uk).

## Ethics Statement

Ethical approval for work conducted in HCS was granted by the East of England—Cambridgeshire and Hertfordshire Research Ethics Committee, reference number 11/EE/0196. Ethical approval for the work conducted in HEAF was received from the National Health Service (NHS) Research Ethics Committee North West-Liverpool East (Reference 12/NW/0500). The patients/participants provided their written informed consent to participate in this study.

## Author Contributions

GB, SD’A, and ED identified the study question. GB and SD’A planned and conducted the statistical analyses. GB, SD’A, and ED wrote the first draft of the paper. All authors (GB, SD’A, CL, AP, IB, JZ, FL, CC, KW, KW-B, and ED) contributed to the writing of subsequent and final drafts of the manuscript. The authors read and approved the final manuscript.

## Funding

This work was funded by the Medical Research Council programme grant (MC_PC_21001 and MC_UU_12011/5) and the Economic and Social Research Council and Medical Research Council jointly Lifelong Health & Wellbeing grants (ES/L002663/1). IB and JZ are supported by the NIHR Southampton BRC, CL is funded by the MRC Versus Arthritis Centre for Musculoskeletal Health and Work (Ref. 22090). The funding bodies played no role in the design of the study or collection, analysis and interpretation of data or in writing the manuscript.

## Conflict of Interest

The authors declare that the research was conducted in the absence of any commercial or financial relationships that could be construed as a potential conflict of interest.

## Publisher’s Note

All claims expressed in this article are solely those of the authors and do not necessarily represent those of their affiliated organizations, or those of the publisher, the editors and the reviewers. Any product that may be evaluated in this article, or claim that may be made by its manufacturer, is not guaranteed or endorsed by the publisher.
